# Comparative effectiveness of pembrolizumab vs. nivolumab in patients with recurrent or advanced NSCLC

**DOI:** 10.1038/s41598-020-70207-7

**Published:** 2020-08-04

**Authors:** Pengfei Cui, Ruixin Li, Ziwei Huang, Zhaozhen Wu, Haitao Tao, Sujie Zhang, Yi Hu

**Affiliations:** 1 Department of Graduate Administration, Chinese PLA General Hospital, Beijing, China; 2 Department of Medical Oncology, Chinese PLA General Hospital, 28 Fuxing Road, Haidian, Beijing, 100853 China; 3 School of Medicine, Nankai University, Tianjin, China

**Keywords:** Cancer immunotherapy, Drug development

## Abstract

The efficacies of pembrolizumab and nivolumab have never been directly compared in a real-world study. Therefore, we sought to retrospectively evaluate the objective response rate (ORR) and the progression-free survival (PFS) of patients with recurrent or advanced non-small cell lung cancer (NSCLC) in a real-world setting. This study included patients with recurrent or advanced NSCLC diagnosed between September 1, 2015 and August 31, 2019, who were treated with programmed cell death 1 (PD-1) inhibitors at the Cancer Center of the Chinese People’s Liberation Army. PFS was estimated for each treatment group using Kaplan–Meier curves and log-rank tests. The multivariate analysis of PFS was performed with Cox proportional hazards regression models. A total of 255 patients with advanced or recurrent NSCLC treated with PD-1 inhibitors were identified. The ORR was significantly higher in the pembrolizumab group than in the nivolumab group, while PFS was not significantly different between the two groups. Subgroup analysis showed that the ORR was significantly higher for pembrolizumab than for nivolumab in patients in the first-line therapy subgroup and in those in the combination therapy as first-line therapy subgroup. Survival analysis of patients receiving combination therapy as second- or further-line therapy showed that nivolumab had better efficacy than pembrolizumab. However, the multivariate analysis revealed no significant difference in PFS between patients treated with pembrolizumab and those treated with nivolumab regardless of the subgroup. In our study, no significant difference in PFS was noted between patients treated with pembrolizumab and those treated with nivolumab in various clinical settings. This supports the current practice of choosing either pembrolizumab or nivolumab based on patient preferences.

## Introduction

Immunotherapy is one of the most important breakthroughs in cancer treatment, and compared with standard therapies, immune checkpoint inhibitors targeting programmed cell death 1 (PD-1) significantly prolong overall survival (OS) in patients with a wide range of tumor types^1,2^. The checkpoint inhibitors nivolumab and pembrolizumab (PD-1 inhibitors) are approved by the Food and Drug Administration (FDA) for use in non-small cell lung cancer (NSCLC); these agents show great efficacy in subsequent-line therapy for advanced NSCLC^3,4^. Although these antibodies are both IgG4 subtype antibodies that target the PD-1 receptor, they bind to different epitopes in the receptor and have different affinities^5^. It is unknown whether the differences in pharmacokinetics and dosing strategies between these two drugs affect the clinical outcomes of patients. In metastatic NSCLC, cross-trial comparisons suggest different efficacies for these two drugs. In a clinical trial, pembrolizumab as a single agent was superior to doublet chemotherapy in patients with high programmed death legend 1 (PD-L1) expression (≥ 50%)^6^. However, nivolumab failed to achieve a positive result in patients with > 1% PD-L1 expression in the following CheckMate 026 study^7^. Although the different outcomes in these two trials could be due to differences in the second-line therapies that patients received in the chemotherapy arms, they do suggest a possible difference in efficacy between these two drugs. Recently, a retrospective real-world analysis of patients with advanced melanoma showed no significant difference in OS between patients treated with first-line pembrolizumab and those treated with first-line nivolumab^8^. A meta-analysis that indirectly compared pembrolizumab and nivolumab as second-line therapy for the treatment of NSCLC also showed no significant differences in OS or progression-free survival (PFS) between pembrolizumab and nivolumab^9^. However, there are currently no real-world studies that have compared the efficacies of these two medications in NSCLC either alone or combined with chemotherapy; therefore, we sought to retrospectively compare the efficacies of pembrolizumab and nivolumab in patients with recurrent or advanced NSCLC in a real-world population to address this gap.

## Results

### Patient characteristics

We included 255 patients with advanced or recurrent NSCLC who were treated with PD-1 inhibitors in our study (consisting of 163 non-squamous NSCLC patients and 92 squamous carcinoma patients). Of the 255 patients, 191 (74.90%) were men, and 64 (25.10%) were women. Their ages ranged from 29 to 86 years, with a median age of 61 years. Patients treated with pembrolizumab were more likely to receive first-line therapy and combination therapy than those treated with nivolumab. There were no differences in age, sex, ECOG performance status, smoking history, disease stage, CNS or intrathoracic metastasis status, histology, mutational status for EGFR or ALK, PD-L1 positivity, treatment cycles or follow-up time between those treated with pembrolizumab and those treated with nivolumab (Table 1).

**Table 1:** Patient demographics.

Characteristic	No. of patients (%)			p value
	All patients (N = 255)	Nivolumab (N = 109)	Pembrolizumab (N = 146)	
Median age (range), years	61 (29–86)	61 (39–83)	60 (35–86)	0.7507
**Sex**				0.56
Male	191 (74.90)	84 (77.06)	107 (73.29)	
Female	64 (25.10)	25 (22.94)	39 (26.71)	
**ECOG performance status**				0.182
0–1	211 (82.75)	86 (78.90)	125 (85.62)	
≥ 2	44 (17.25)	23 (21.10)	21 (14.38)	
**Smoking history**				0.605
Current or former	157 (61.57)	65 (59.63)	92 (63.01)	
Never	98 (38.43)	44 (40.37)	54 (36.99)	
**Stage**				0.126
Recurrence	32 (12.55)	18 (16.51)	14 (9.59)	
IIIB–IV	223 (87.45)	91 (83.49)	132 (90.41)	
**Metastasis**				
CNS versus no CNS	57 (22.35) versus 198 (77.65)	27 (24.77) versus 82 (75.23)	30 (20.55) versus 116 (79.45)	0.45
Intrathoracic only versus no Intrathoracic	123 (48.24) versus 132 (51.76)	59 (54.13) versus 50 (45.87)	64 (43.84) versus 82 (56.16)	0.128
**Histology**				0.148
Squamous	92 (36.08)	45 (41.28)	47 (32.19)	
Nonsquamous	163 (63.92)	64 (58.72)	99 (67.81)	
**EGFR mutation status**				0.054
Positive	35 (13.73)	16 (14.68)	19 (13.01)	
Negative	136 (53.33)	49 (44.95)	87 (59.59)	
Not examined	84 (32.94)	44 (40.37)	40 (27.40)	
**ALK fusion status**				0.177
Positive	4 (1.57)	3 (2.75)	1 (0.68)	
Negative	188 (73.73)	75 (68.81)	113 (77.40)	
Not examined	63 (24.71)	31 (28.44)	32 (21.92)	
**PD-L1 expression**				0.055
< 1	15 (5.88)	6 (5.50)	9 (6.16)	
1–49	30 (11.76)	12 (11.01)	18 (12.33)	
≥ 50	35 (13.73)	8 (7.34)	27 (18.49)	
Not examined	175 (68.63)	83 (76.15)	92 (63.01)	
**Treatment lines**				0.007
1	85 (33.33)	26 (23.85)	59 (40.41)	
≥ 2	170 (66.67)	83 (76.15)	87 (59.59)	
**Combined with chemotherapy**				0.000
No	143 (56.08)	78 (71.56)	65 (44.52)	
Yes	112 (43.92)	31 (28.44)	81 (55.48)	
Cycles of treatment, median (range), No	5 (1–38)	6 (1–38)	5 (1–24)	0.1123
Follow up time, (range), days	249 (13–1,288)	289 (31–1,288)	230 (28–1,286)	0.2035
Overall response rate	87 (34.12)	25 (22.94)	62 (42.47)	0.001

*ECOG* Eastern Cooperative Oncology Group, *CNS* central nervous system, *EGFR* epidermal growth factor receptor gene, *ALK* anaplastic lymphoma kinase gene, *PD-L1* programmed cell death ligand 1, *No.* number.

### PFS of patients with recurrent or advanced NSCLC treated with pembrolizumab vs. nivolumab

**Figure 1:**
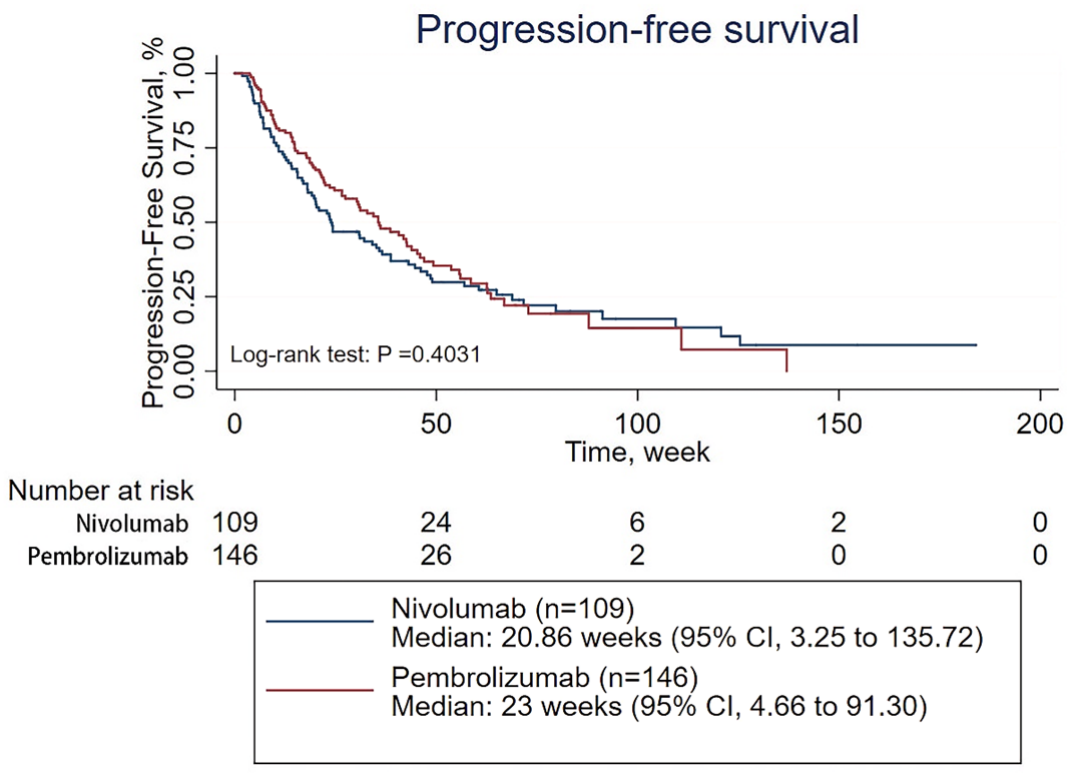
Progression-free survival of patients with NSCLC treated with pembrolizumab or nivolumab.

With a median follow-up time of 249 days, the median PFS time for all treated patients was 22.14 weeks [95% confidence interval (CI) 3.83–116.77]. The median PFS time for patients treated with pembrolizumab was 23 weeks (95% CI 4.66–91.30), while the median PFS time for those treated with nivolumab was 20.86 weeks (95% CI 3.25–135.72) (Fig. 1). The survival analysis showed that the ORR was significantly higher in the patients treated with pembrolizumab than in those treated with nivolumab (62 of 146 patients [42.47%] vs 25 of 109 patients [22.94%]; p = 0.001). However, there was no significant difference in PFS between the patients treated with pembrolizumab and those treated with nivolumab (p = 0.4031) (Fig. 1). When adjusted for age, sex, number of treatment lines, PD-L1 expression, CNS metastasis status, histology, pretreatment ECOG performance status, disease stage, treatment cycles, and therapeutic strategy (combined with chemotherapy or not), which may affect the efficacy of the PD-1 inhibitors, the multivariate analysis revealed no significant difference in PFS between the patients treated with pembrolizumab and those treated with nivolumab (HR 0.917; 95% CI 0.663–1.267; p = 0.598) (Table 2, Supplementary Table 7).

**Table 2:** Hazard ratio for progression-free survival (PFS) for receiving pembrolizumab versus nivolumab.

	Univariable hazard ratio (95% CI)	p value	Multivariable hazard ratio (95% CI)	p value
**NSCLC**				
PFS	0.877 (0.645–1.192)	0.402	0.917 (0.663–1.267)	0.598
**NSCLC in the first line**				
PFS	0.986 (0.490–1.984)	0.968	0.886 (0.641–1.224)	0.462
**NSCLC receiving monotherapy in the first line**				
PFS	0.737 (0.233–2.336)	0.605	2.410 (0.141–41.276)	0.544
**NSCLC receiving combined therapy in the first line**				
PFS	1.247 (0.458–3.397)	0.666	3.494 (0.904–13.507)	0.07
**NSCLC in the second line**				
PFS	0.956 (0.675–1.352)	0.798	0.884 (0.612–1.278)	0.513
**NSCLC receiving monotherapy in the second line**				
PFS	0.793 (0.521–1.206)	0.278	0.772 (0.497–1.200)	0.25
**NSCLC receiving combined therapy in the second line**				
PFS	2.043 (1.004–4.154)	0.049	2.396 (0.788–7.283)	0.124

*CI* confidence interval, *NSCLC* non-small cell lung cancer.

### Subgroup analyses of PFS between pembrolizumab- and nivolumab-treated patients

**Figure 2:**
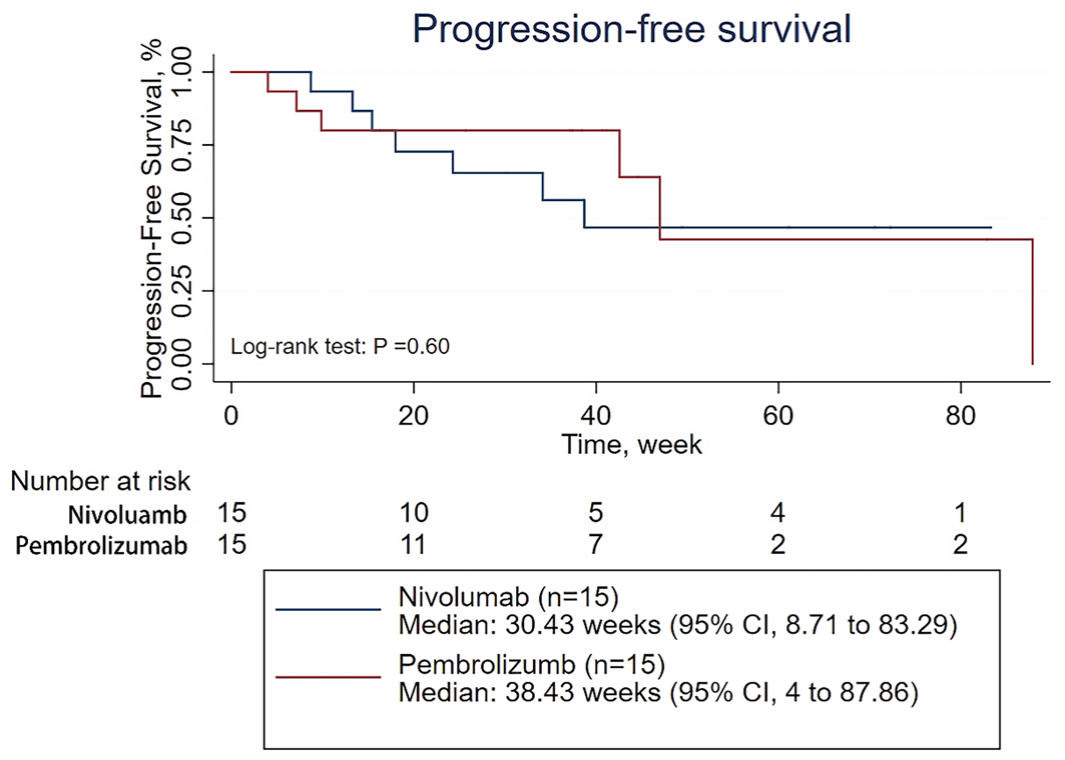
Progression-free survival for patients receiving PD-1 inhibitors monotherapy in the first line therapy.

**Figure 3:**
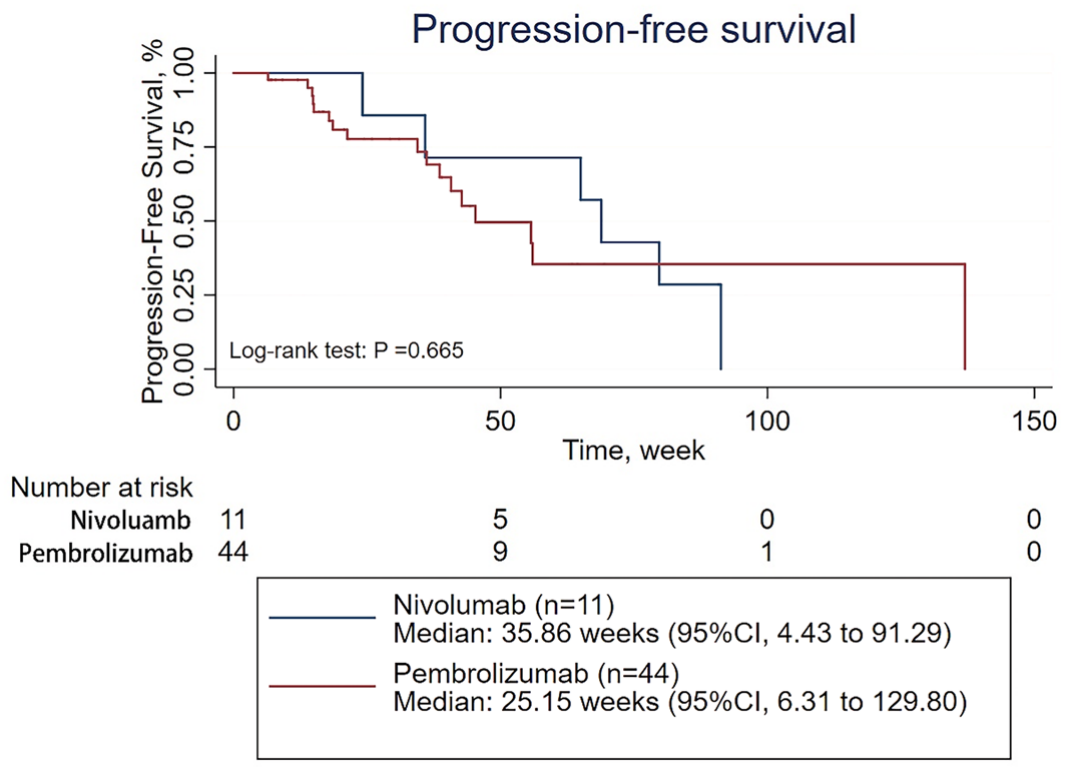
Progression-free survival for patients receiving combined therapy in the first line therapy.

**Figure 4:**
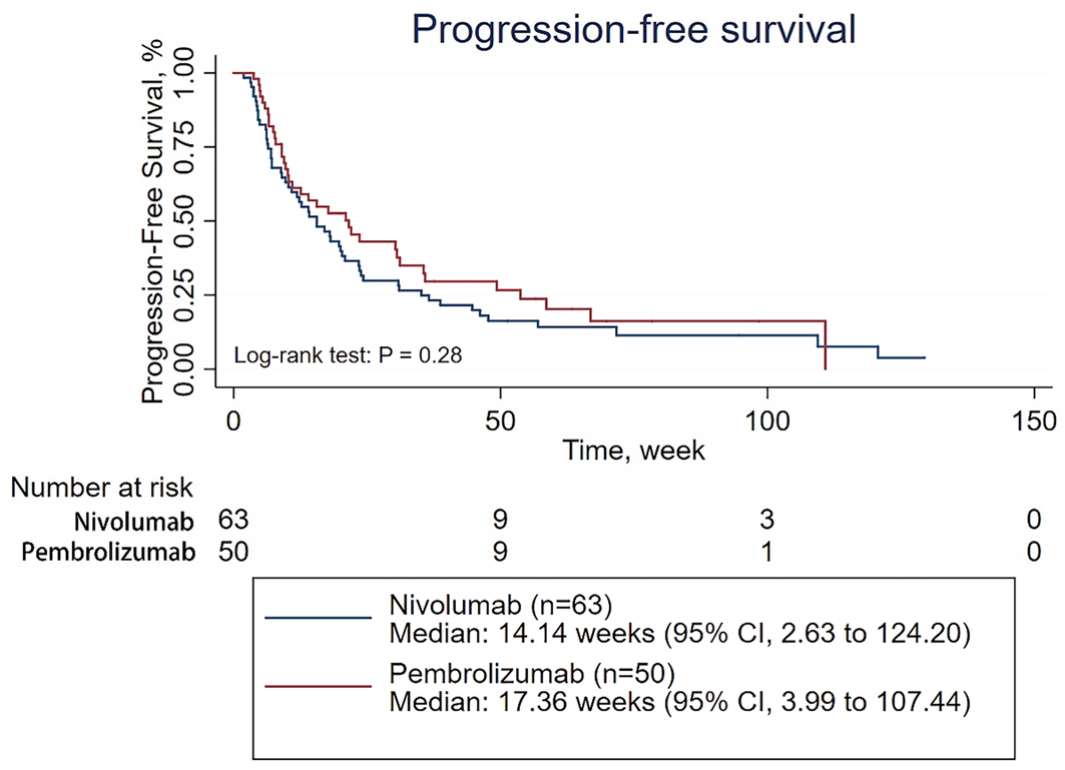
Progression-free survival of for patients receiving PD-1 inhibitors monotherapy in the second line therapy.

**Figure 5:**
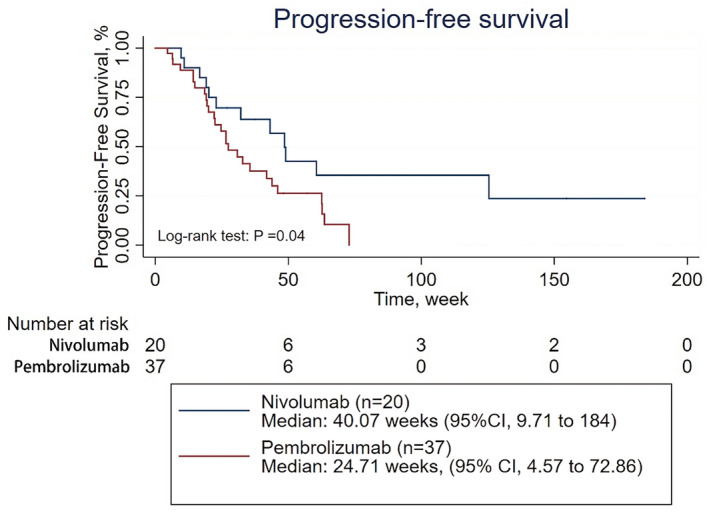
Progression-free survival of for patients receiving combined therapy in the second line therapy.

Then, subgroup analyses of PFS were conducted on patients treated with first-line therapy, patients receiving PD-1 inhibitor monotherapy as their first-line therapy, patients receiving combination therapy as their first-line therapy, patients treated with second-line therapy, patients receiving PD-1 inhibitor monotherapy as second- or further-line therapy, and patients receiving combination therapy as second- or further-line therapy (Supplementary Tables S1–S6). The survival analysis showed that the ORR was significantly higher in patients treated with pembrolizumab than in those treated with nivolumab among patients in the first-line therapy subgroup and among patients in the receiving combination therapy as their first-line therapy subgroup (39 of 59 patients [66.10%] vs 8 of 26 patients [30.77%], p = 0.004; 33 of 44 patients [75.00%] vs 2 of 11 patients [18.18%], p = 0.001;, respectively) (Supplementary Tables S1 and S3). However, there was no significant difference in PFS between the patients treated with pembrolizumab and those treated with nivolumab regardless of the subgroup, except for the subgroup of patients receiving combination therapy as second- or further-line therapy, in which nivolumab demonstrated better efficacy than pembrolizumab (p = 0.04) (Figs. 2, 3, 4, 5 and Supplementary Figs. S1 and S2). When adjusted for age, sex, PD-L1 expression, CNS metastasis status, histology, pretreatment ECOG performance status, disease stage, treatment cycles, and combination status (combined with single-agent chemotherapy or double-agent chemotherapy), the multivariate analysis revealed no significant difference in PFS between patients treated with pembrolizumab and those treated with nivolumab regardless of the subgroup (Table 2, Supplementary Tables S8–S13).

## Discussion

Pembrolizumab and nivolumab are both PD-1 receptor inhibitors whose efficacies have been demonstrated in several clinical trials^10–13^. Both have been approved for the treatment of NSCLC and melanoma by the FDA. Because there is no head-to-head randomized controlled trial (RCT) on the differences between pembrolizumab and nivolumab to aid in therapeutic choices, this study compared the efficacies of these two inhibitors.

First, we compared pembrolizumab to nivolumab in patients with recurrent or advanced NSCLC. The survival analysis showed that pembrolizumab had a better ORR than nivolumab, but no significant difference in PFS was found between pembrolizumab and nivolumab. As the treatment lines and combination status were significantly different between the two groups, we conducted subgroup analyses according to the treatment lines and combination status. The first-line therapy subgroup analysis also suggested that pembrolizumab had a better ORR than nivolumab, while the second-line therapy subgroup analysis did not. PFS was not significantly different between the two PD-1 inhibitors. Since the combination status was significantly different between pembrolizumab and nivolumab in both the first-line and second-line therapy subgroup analyses, further subgroup analysis was conducted. The monotherapy subgroup analysis for first-line and second-line therapy revealed no significant differences in the ORR or PFS. The combination status is believed to affect efficacy in patients, so we divided the chemotherapy combined with an anti-PD-1 agent subgroup into two categories: single-agent chemotherapy and double-agent chemotherapy. Fortunately, the combination status was balanced between the two anti-PD-1 agents for first-line and second-line therapy. The combination subgroup analysis for first-line therapy showed that pembrolizumab had a better ORR than nivolumab, while no significant difference in PFS was found between the two groups. The combination subgroup analysis for second-line therapy showed that nivolumab was associated with longer PFS than pembrolizumab, while no significant difference in the ORR was found between the two groups. However, when adjusted for age, sex, PD-L1 expression, CNS metastasis status, histology, pretreatment ECOG performance status, disease stage, treatment cycles, and combination therapy status, the multivariate analysis revealed no significant difference between the two anti-PD-1 agent groups. These results are similar to the findings of the latest meta-analysis^9^.

To our knowledge, this is the first cohort study that compared treatment outcomes between pembrolizumab and nivolumab in patients with recurrent or advanced NSCLC. However, there are several limitations to our study. First, this is a single-center retrospective study of NSCLC patients, and information bias cannot be excluded; thus, these results warrant further study with larger cohorts. Second, the follow-up time was not long enough to allow us to fully address long-term survival outcomes. Third, although approved by the FDA, the test kits used to assess PD-L1 expression in this study were different for pembrolizumab and nivolumab, which may also affect our results.

## Conclusions

According to our comparisons, pembrolizumab and nivolumab demonstrated similar survival benefits in patients with recurrent or advanced NSCLC in various clinical settings. Therefore, our study may support the current clinical practice of choosing either drug based on patient and clinician preferences.

## Methods

We conducted a real-world, retrospective study to compare the effectiveness of pembrolizumab and nivolumab in patients with recurrent or advanced NSCLC. The Ethics Committee of the Chinese People’s Liberation Army General Hospital approved this retrospective cohort study on patients with advanced (stage IIIB to IV) or recurrent NSCLC who were treated with PD-1 inhibitors at the Cancer Center of the Chinese People’s Liberation Army. Patients who received at least one cycle of nivolumab or pembrolizumab and completed at least one follow-up visit were included. The end of the follow-up period was August 31, 2019. We reviewed patient medical records from September 1, 2015 to August 31, 2019. From this review, we identified 255 patients who received nivolumab or pembrolizumab. Both PD-1 inhibitor monotherapy and combination therapy (PD-1 inhibitor plus chemotherapy) were included. The monotherapies included PD-1 inhibitors for NSCLC. The combination therapies included PD-1 inhibitors + pemeterxed + carboplatin for non-squamous NSCLC, PD-1 inhibitors + carboplatin + nab-paclitaxel for lung squamous carcinoma, PD-1 inhibitors + pemeterxed for non-squamous NSCLC, and PD-1 inhibitors + nab-paclitaxel for lung squamous carcinoma depending on the choice of the clinician. Medical records were reviewed, and data on clinicopathological features and treatment history were extracted. We collected the following data: patient demographics, therapeutic regimen, type of disease, stage of disease, time point of disease progression, driver gene mutation status, and PD-L1 expression. We tested PD-L1 expression using 2 FDA-approved clinical immunohistochemical markers for PD-L1 expression: Dako PD-L1 22C3 pharmDx for pembrolizumab and Dako PD-L1 28-8 pharmDx for nivoluamb. Tumor response was assessed by computed tomography performed every 6 to 8 weeks and evaluated according to the Response Evaluation Criteria in Solid Tumors (RECIST) version 1.1. PFS was measured from the time of treatment initiation to clinical or radiographic disease progression or death from any cause. Patients without documented clinical or radiographic progression were censored on the date of the last follow-up. Disease progression was defined according to immune-related response criteria^14^. The current study was approved by the Institutional Review Board of People's Liberation Army General Hospital, Beijing, China (approval number: S2018-092-01). This clinical study was conducted in accordance with the Helsinki Declaration. Because of the retrospective nature of the study, informed consent was waived by the Ethics Committee of Chinese People’s Liberation Army General Hospital. This paper does not contain any individual person’s data in any form.

### Statistical analysis

Statistical analysis was performed using Stata version 15.1 (StataCorp). To compare groups, we used Fisher’s exact test (categorical variables) or the Wilcoxon rank-sum test (continuous variables). Fisher’s exact test was applied to assess the objective response rate (ORR). PFS was compared between patients treated with pembrolizumab and those treated with nivolumab using Kaplan–Meier curves and log-rank analysis. Cox regression analysis was also used to compare PFS between these populations. Univariate and multivariate Cox proportional hazards regression models were adopted to determine hazard ratios (HRs). The multivariate analysis was performed with adjustments for age, sex, number of treatment lines, PD-L1 expression, central nervous system (CNS) metastasis status, histology, pretreatment Eastern Cooperative Oncology Group (ECOG) performance status, disease stage, treatment cycles, combination therapy status, and combination with single-agent chemotherapy or double-agent chemotherapy. All P values were based on a 2-sided hypothesis, and those less than 0.05 were considered statistically significant.

## Supplementary information

Supplementary Information 1.

Supplementary Information 2.

## Data Availability

The data used to support the findings of this study are available from the corresponding author upon request.
