# Supplementary material for: Comparative effectiveness of pembrolizumab vs. nivolumab in patients with recurrent or advanced NSCLC
Source: Sci Rep. 2020 Aug 4;10:13160. doi: 10.1038/s41598-020-70207-7 (PMC7403144; doi:10.1038/s41598-020-70207-7)

**Comparative effectiveness of pembrolizumab vs. nivolumab in patients with** **recurrent or advanced NSCLC**

Pengfei Cui^a,b#^; Ruixin Li^b#^; Ziwei Huang^c,b#^; Zhaozhen Wu^c,b^; Haitao Tao^b^; Sujie Zhang^b^; Yi Hu^b,a,c*^

^a^Department of Graduate Administration, Chinese PLA General Hospital, Beijing, China

^b^Department of Medical Oncology, Chinese PLA General Hospital, Beijing, China

^c^School of Medicine, Nankai University, Tianjin, China

^#^These authors contributed equally to this paper.

***Corresponding author**

Prof. Yi Hu, Department of Medical Oncology, Chinese PLA General Hospital, 28 Fuxing Road, Haidian, Beijing 100853, China

Phone: (+86) 13911031186

E-mail: huyi_0912@126.com

Figure S1. Progression-free survival for patients in the first line therapy.


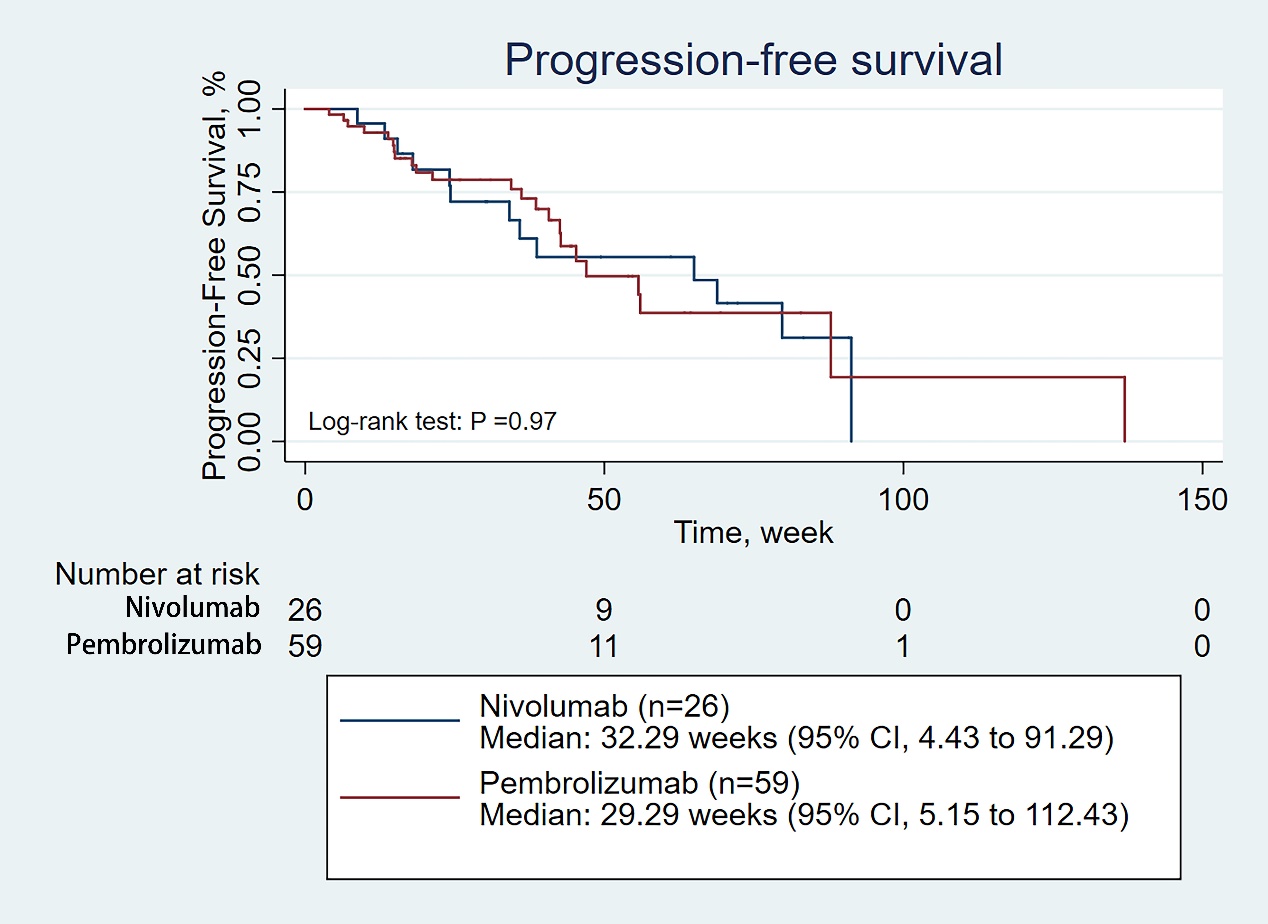


Figure S2. Progression-free survival of for patients in the second line therapy.


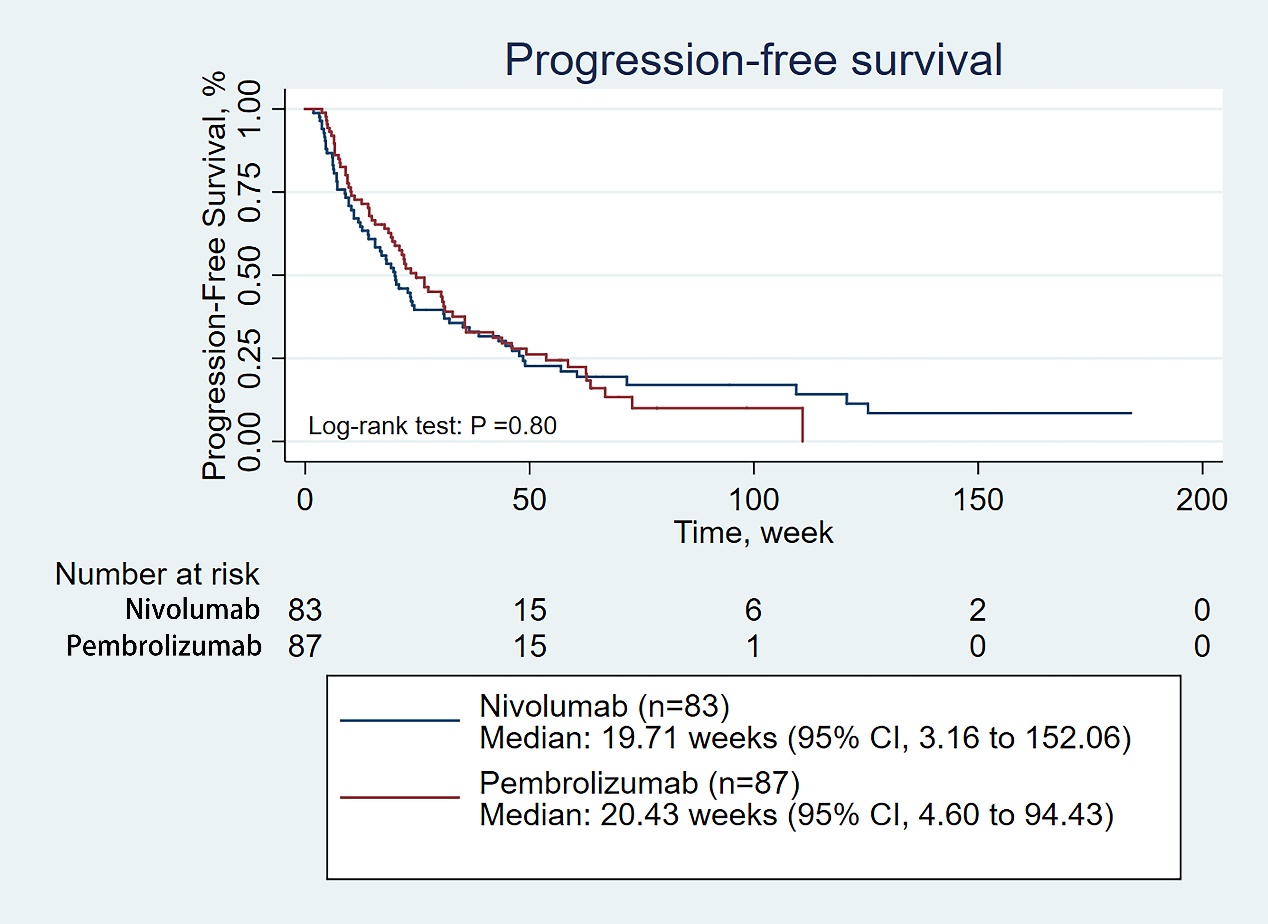

Supplement: Supplementary file 1 — Supplementary Information 1. [file 41598_2020_70207_MOESM1_ESM.docx]
