# Supplementary material for: Comparative effectiveness of pembrolizumab vs. nivolumab in patients with recurrent or advanced NSCLC
Source: Sci Rep. 2020 Aug 4;10:13160. doi: 10.1038/s41598-020-70207-7 (PMC7403144; doi:10.1038/s41598-020-70207-7)
Supplement: Supplementary file 2 — Supplementary Information 2. [file 41598_2020_70207_MOESM2_ESM.docx]

**Comparative effectiveness of pembrolizumab vs. nivolumab in patients with** **recurrent or advanced NSCLC**

Pengfei Cui^a,b#^; Ruixin Li^b#^; Ziwei Huang^c,b#^; Zhaozhen Wu^c,b^; Haitao Tao^b^; Sujie Zhang^b^; Yi Hu^b,a,c*^

^a^Department of Graduate Administration, Chinese PLA General Hospital, Beijing, China

^b^Department of Medical Oncology, Chinese PLA General Hospital, Beijing, China

^c^School of Medicine, Nankai University, Tianjin, China

^#^These authors contributed equally to this paper.

***Corresponding author**

Prof. Yi Hu, Department of Medical Oncology, Chinese PLA General Hospital, 28 Fuxing Road, Haidian, Beijing 100853, China

Phone: (+86) 13911031186

E-mail: huyi_0912@126.com

Table S1. Patient demographics for patients in the first line therapy.

|  | No. of patients (%) | | |  |
| --- | --- | --- | --- | --- |
| Characteristic | All patients (N=85) | Nivolumab （N=26） | Pembrolizumab （N=59） | P value |
| Median age (range), years | 62(35-79) | 66(45-79) | 61(35-79) | 0.248 |
| Sex |  |  |  | 0.553 |
| Male | 69(81.18) | 20(76.92) | 49(83.05) |  |
| Female | 16(18.82) | 6(23.08) | 10(16.95) |  |
| ECOG performance status |  |  |  | 0.193 |
| 0–1 | 78(91.76) | 22(84.62) | 56(94.92) |  |
| ≥2 | 7(8.24) | 4(15.38) | 3(5.08) |  |
| Smoking history |  |  |  | 0.12 |
| Current or former | 60(70.59) | 15(57.69) | 45(76.27) |  |
| Never | 25(29.41) | 11(42.31) | 14(23.73) |  |
| Stage |  |  |  | 0.696 |
| Recurrence | 8(9.41) | 3(11.54) | 5(8.47) |  |
| IIIB–IV | 77(90.59) | 23(88.46) | 54(91.53) |  |
| Metastasis |  |  |  |  |
| CNS versus no CNS | 10(11.76) versus 75(88.24) | 1(3.85) versus 25(96.15) | 9(15.25) versus 50(84.75) | 0.166 |
| Intrathoracic only versus no Intrathoracic | 27(31.76) versus 58(68.24) | 9(34.62) versus 17(65.38) | 18(30.51) versus 41(69.49) | 0.802 |
| Histology |  |  |  | 0.241 |
| Squamous | 40(47.06) | 15(57.69) | 25(42.37) |  |
| Nonsquamous | 45(52.94) | 11(42.31) | 34(57.63) |  |
| PD-L1 expression |  |  |  | 0.693 |
| ＜1 | 9(10.59) | 3(11.54) | 6(10.17) |  |
| 1-49 | 18(21.18) | 5(19.23) | 13(22.03) |  |
| ≥50 | 16(18.82) | 3(11.54) | 13(22.03) |  |
| Not examined | 42(49.41) | 15(57.69) | 27(45.76) |  |
| Combined with chemotherapy |  |  |  | 0.006 |
| No | 30(35.29) | 15(57.69) | 15(25.42) |  |
| Yes | 55(64.71) | 11(42.31) | 44(74.58) |  |
| Cycles of treatment, median(range), No. | 6(1-31) | 8(1-31) | 6(2-22) | 0.0381 |
| Follow up time,(range), days | 260(28-1286) | 310.5(31-744) | 225(28-1286) | 0.215 |
| Overall response rate | 47(55.29) | 8(30.77) | 39(66.10) | 0.004 |

ECOG, Eastern Cooperative Oncology Group; CNS, central nervous system; PD-L1, programmed cell death ligand 1; No., number.

Table S2. Patient demographics for patients receiving PD-1 inhibitors monotherapy in the first line therapy.

|  | No. of patients (%) | | |  |
| --- | --- | --- | --- | --- |
| Characteristic | All patients (N=30) | Nivolumab （N=15） | Pembrolizumab （N=15） | P value |
| Median age (range), years | 63.5(45-79) | 67(45-79) | 62(56-79) | 0.9337 |
| Sex |  |  |  | 1.000 |
| Male | 23(76.67) | 12(80.00) | 11(73.33) |  |
| Female | 7(23.33) | 3(20.00) | 4(26.67) |  |
| ECOG performance status |  |  |  | 0.598 |
| 0–1 | 26(86.67) | 12(80.00) | 14(93.33) |  |
| ≥2 | 4(13.33) | 3(20.00) | 1(6.67) |  |
| Smoking history |  |  |  | 0.71 |
| Current or former | 18(60.00) | 8(53.33) | 10(66.67) |  |
| Never | 12(40.00) | 7(46.67) | 5(33.33) |  |
| Stage |  |  |  | 0.483 |
| Recurrence | 2(6.67) | 2(13.33) | 0(0.00) |  |
| IIIB–IV | 28(93.33) | 13(86.67) | 15(100.00) |  |
| Metastasis |  |  |  |  |
| CNS versus no CNS | 3(10.00) versus 27(90.00) | 0(0) versus 15(100.00) | 3(20.00) versus 12(80.00) | 0.224 |
| Intrathoracic only versus no Intrathoracic | 12(40.00) versus 18(60.00) | 6(40.00) versus 9(60.00) | 6(40.00) versus 9(60.00) | 1.000 |
| Histology |  |  |  | 0.272 |
| Squamous | 14(46.67) | 9(60.00) | 5(33.33) |  |
| Nonsquamous | 16(53.33) | 6(40.00) | 10(66.67) |  |
| PD-L1 expression |  |  |  | 0.435 |
| ＜1 | 4(13.33) | 3(20.00) | 1(6.67) |  |
| 1-49 | 3(10.00) | 2(13.33) | 1(6.67) |  |
| ≥50 | 8(26.67) | 2(13.33) | 6(40.00) |  |
| Not examined | 15(50.00) | 8(53.33) | 7(46.67) |  |
| Cycles of treatment, median(range), No. | 8(2-31) | 8(2-31) | 8(2-22) | 0.2588 |
| Follow up time,(range), days | 277(28-1071) | 300(74-697) | 269(28-1071) | 0.8195 |
| Overall response rate | 12(40.00) | 6(40) | 6(40) | 1.000 |

ECOG, Eastern Cooperative Oncology Group; CNS, central nervous system; PD-L1, programmed cell death ligand 1; No., number.

Table S3. Patient demographics for patients receiving combined therapy in the first line therapy.

|  | No. of patients (%) | | |  |
| --- | --- | --- | --- | --- |
| Characteristic | All patients (N=55) | Nivolumab （N=11） | Pembrolizumab （N=44） | P value |
| Median age (range), years | 62(35-78) | 66(47-78) | 61(35-77) | 0.2688 |
| Sex |  |  |  | 0.362 |
| Male | 46(83.64) | 8(72.73) | 38(86.36) |  |
| Female | 9(16.36) | 3(27.27) | 6(13.64) |  |
| ECOG performance status |  |  |  | 0.495 |
| 0–1 | 52(94.55) | 10(90.91) | 42(95.45) |  |
| ≥2 | 3(5.45) | 1(9.09) | 2(4.55) |  |
| Smoking history |  |  |  | 0.427 |
| Current or former | 42(76.36) | 7(63.64) | 35(79.55) |  |
| Never | 13(23.64) | 4(36.36) | 9(20.45) |  |
| Stage |  |  |  | 1.000 |
| Recurrence | 6(10.91) | 1(9.09) | 5(11.36) |  |
| IIIB–IV | 49(89.09) | 10(90.91) | 39(88.64) |  |
| Metastasis |  |  |  |  |
| CNS versus no CNS | 7(12.73) versus 48(87.27) | 1(9.09) versus 10(90.91) | 6(13.64) versus 38(86.36) | 1.000 |
| Intrathoracic only versus no Intrathoracic | 15(27.27) versus 40(72.73) | 3(27.27) versus 8(72.73) | 12(27.27) versus 32(72.73) | 1.000 |
| Histology |  |  |  | 0.739 |
| Squamous | 26(47.27) | 6(54.55) | 20(45.45) |  |
| Nonsquamous | 29(52.73) | 5(45.45) | 24(54.55) |  |
| PD-L1 expression |  |  |  | 0.748 |
| ＜1 | 5(9.09) | 0(0) | 5(11.36) |  |
| 1-49 | 15(27.27) | 3(27.27) | 12(27.27) |  |
| ≥50 | 8(14.55) | 1(9.09) | 7(15.91) |  |
| Not examined | 27(49.09) | 7(63.64) | 20(45.45) |  |
| Cycles of treatment, median(range), No. | 5(1-18) | 6(1-17) | 5(2-18) | 0.6035 |
| Combined status |  |  |  | 0.674 |
| Single chemotherapy | 11(20.00) | 3(27.27) | 8(18.18) |  |
| Double chemotherapy | 44(80.00) | 8(72.73) | 36(81.82) |  |
| Follow up time,(range), days | 205(31-1286) | 321(31-744) | 193.5(44-1286) | 0.5142 |
| Overall response rate | 35(63.64) | 2(18.18) | 33(75.00) | 0.001 |

ECOG, Eastern Cooperative Oncology Group; CNS, central nervous system; PD-L1, programmed cell death ligand 1; No., number.

Table S4. Patient demographics for patients in the second line therapy.

|  | No. of patients (%) | | |  |
| --- | --- | --- | --- | --- |
| Characteristic | All patients (N=170) | Nivolumab （N=83） | Pembrolizumab （N=87） | P value |
| Median age (range), years | 60(29-86) | 60(29-83) | 59(35-86) | 0.7993 |
| Sex |  |  |  | 0.173 |
| Male | 122(71.76) | 64(77.11) | 58(66.67) |  |
| Female | 48(28.24) | 19(22.89) | 29(33.33) |  |
| ECOG performance status |  |  |  | 0.853 |
| 0–1 | 133(78.24) | 64(77.11) | 69(79.31) |  |
| ≥2 | 37(21.76) | 19(22.89) | 18(20.69) |  |
| Smoking history |  |  |  | 0.441 |
| Current or former | 97(57.06) | 50(60.24) | 47(54.02) |  |
| Never | 73(42.94) | 33(39.76) | 40(45.98) |  |
| Stage |  |  |  | 0.187 |
| Recurrence | 24(14.12) | 15(18.07) | 9(10.34) |  |
| IIIB–IV | 146(85.88) | 68(81.93) | 78(89.66) |  |
| Metastasis |  |  |  |  |
| CNS versus no CNS | 47(27.65) versus 123(72.35) | 26(31.33) versus 57(68.67) | 21(24.14) versus 66(75.86) | 0.309 |
| Intrathoracic only versus no Intrathoracic | 96(56.47) versus 74(43.53) | 50(60.24) versus 33(39.76) | 46(52.87) versus 41(47.13) | 0.356 |
| Histology |  |  |  | 0.137 |
| Squamous | 52(30.59) | 30(36.14) | 22(25.29) |  |
| Nonsquamous | 118(69.41) | 53(63.86) | 65(74.71) |  |
| EGFR mutation status |  |  |  | 0.478 |
| Positive | 35(20.59) | 16(19.28) | 19(21.84) |  |
| Negative | 83(48.82) | 38(45.78) | 45(51.72) |  |
| Not examined | 52(30.59) | 29(34.94) | 23(26.44) |  |
| ALK fusion status |  |  |  | 0.343 |
| Positive | 4(2.35) | 3(3.61) | 1(1.15) |  |
| Negative | 124(72.94) | 57(68.67) | 67(77.01) |  |
| Not examined | 42(24.71) | 23(27.71) | 19(21.84) |  |
| PD-L1 expression |  |  |  | 0.185 |
| ＜1 | 6(3.53) | 3(3.61) | 3(3.45) |  |
| 1-49 | 12(7.06) | 7(8.43) | 5(5.75) |  |
| ≥50 | 19(11.18) | 5(6.02) | 14(16.09) |  |
| Not examined | 133(78.24) | 68(81.93) | 65(74.71) |  |
| Combined with chemotherapy |  |  |  | 0.015 |
| No | 113(66.47) | 63(75.90) | 50(57.47) |  |
| Yes | 57(33.53) | 20(24.10) | 37(42.53) |  |
| Cycles of treatment, median(range), No. | 4.5(1-38) | 5(1-38) | 4(1-24) | 0.3233 |
| Follow up time,(range), days | 248(13-1288) | 287(13-1288) | 230(35-1020) | 0.4394 |
| Overall response rate | 40(23.53) | 17(20.48) | 23(26.44) | 0.373 |

ECOG, Eastern Cooperative Oncology Group; CNS, central nervous system; EGFR, epidermal growth factor receptor gene; ALK, anaplastic lymphoma kinase gene; PD-L1, programmed cell death ligand 1; No., number.

Table S5. Patient demographics for patients receiving PD-1 inhibitors monotherapy in the second line therapy.

|  | No. of patients (%) | | |  |
| --- | --- | --- | --- | --- |
| Characteristic | All patients (N=113) | Nivolumab （N=63） | Pembrolizumab （N=50） | P value |
| Median age (range), years | 60(29-83) | 61(29-83) | 60(36-78) | 0.3886 |
| Sex |  |  |  | 0.211 |
| Male | 80(29.20) | 48(76.19) | 32(64.00) |  |
| Female | 33(29.20) | 15(23.81) | 18(36.00) |  |
| ECOG performance status |  |  |  | 0.829 |
| 0–1 | 85(75.22) | 48(76.19) | 37(74.00) |  |
| ≥2 | 28(24.78) | 15(23.81) | 13(26.00) |  |
| Smoking history |  |  |  | 0.849 |
| Current or former | 61(53.98) | 35(55.56) | 26(52.00) |  |
| Never | 52(46.02) | 28(44.44) | 24(48.00) |  |
| Stage |  |  |  | 0.081 |
| Recurrence | 20(17.70) | 15(23.81) | 5(10.00) |  |
| IIIB–IV | 93(82.30) | 48(76.19) | 45(90.00) |  |
| Metastasis |  |  |  |  |
| CNS versus no CNS | 34(30.09) versus 79(69.91) | 18(28.57) versus 45(71.43) | 16(32.00) versus 34(68.00) | 0.837 |
| Intrathoracic only versus no Intrathoracic | 74(65.49) versus 39(34.51) | 45(71.43) versus 18(28.57) | 29(58.00) versus 21(42.00) | 0.165 |
| Histology |  |  |  | 0.219 |
| Squamous | 35(30.97) | 23(36.51) | 12(24.00) |  |
| Nonsquamous | 78(69.03) | 40(63.49) | 38(76.00) |  |
| EGFR mutation status |  |  |  | 0.725 |
| Positive | 23(20.35) | 11(17.46) | 12(24.00) |  |
| Negative | 55(48.67) | 32(50.79) | 23(46.00) |  |
| Not examined | 35(30.97) | 20(31.75) | 15(30.00) |  |
| ALK fusion status |  |  |  | 0.928 |
| Positive | 3(2.65) | 2(3.17) | 1(2.00) |  |
| Negative | 81(71.68) | 44(69.84) | 37(74.00) |  |
| Not examined | 29(25.66) | 17(26.98) | 12(24.00) |  |
| PD-L1 expression |  |  |  | 0.232 |
| ＜1 | 4(3.54) | 2(3.17) | 2(4.00) |  |
| 1-49 | 8(7.08) | 6(9.52) | 2(4.00) |  |
| ≥50 | 8(7.08) | 2(3.17) | 6(12.00) |  |
| Not examined | 93(82.30) | 53(84.13) | 40(80.00) |  |
| Cycles of treatment, median(range), No. | 4(1-38) | 4(1-38) | 4(1-24) | 0.5893 |
| Follow up time,(range), days | 216(13-1225) | 237(13-1225) | 208(35-1020) | 0.7836 |
| Overall response rate | 16(14.16) | 9(14.29) | 7(14.00) | 1.000 |

ECOG, Eastern Cooperative Oncology Group; CNS, central nervous system; EGFR, epidermal growth factor receptor gene; ALK, anaplastic lymphoma kinase gene; PD-L1, programmed cell death ligand 1; No., number.

Table S6. Patient demographics for patients receiving combined therapy in the second line therapy.

|  | No. of patients (%) | | |  |
| --- | --- | --- | --- | --- |
| Characteristic | All patients (N=57) | Nivolumab （N=20） | Pembrolizumab （N=37） | P value |
| Median age (range), years | 56(35-86) | 58.5(36-69) | 56(35-86) | 0.7696 |
| Sex |  |  |  | 0.537 |
| Male | 42(73.68) | 16(80.00) | 26(70.27) |  |
| Female | 15(26.32) | 4(20.00) | 11(29.73) |  |
| ECOG performance status |  |  |  | 0.705 |
| 0–1 | 48(84.21) | 16(80.00) | 32(86.49) |  |
| ≥2 | 9(15.79) | 4(20.00) | 5(13.51) |  |
| Smoking history |  |  |  | 0.251 |
| Current or former | 36(63.16) | 15(75.00) | 21(56.76) |  |
| Never | 21(36.84) | 5(25.00) | 16(43.24) |  |
| Stage |  |  |  | 0.286 |
| Recurrence | 4(7.02) | 0(0.00) | 4(10.81) |  |
| IIIB–IV | 53(92.98) | 20(100) | 33(89.19) |  |
| Metastasis |  |  |  |  |
| CNS versus no CNS | 13(22.81) versus 44(77.19) | 8(40.00) versus 12(60.00) | 5(13.51) versus 32(86.49) | 0.044 |
| Intrathoracic only versus no Intrathoracic | 22(38.60) versus 35(61.40) | 5(25.00) versus 15(75.00) | 17(45.95) versus 20(54.05) | 0.159 |
| Histology |  |  |  | 0.557 |
| Squamous | 17(29.82) | 7(35.00) | 10(27.03) |  |
| Nonsquamous | 40(70.18) | 13(65.00) | 27(72.97) |  |
| EGFR mutation status |  |  |  | 0.101 |
| Positive | 12(21.05) | 5(25.00) | 7(18.92) |  |
| Negative | 28(49.12) | 6(30.00) | 22(59.46) |  |
| Not examined | 17(29.82) | 9(45.00) | 8(21.62) |  |
| ALK fusion status |  |  |  | 0.236 |
| Positive | 1(1.75) | 1(5.00) | 0(0.00) |  |
| Negative | 43(75.44) | 13(65.00) | 30(81.08) |  |
| Not examined | 13(22.81) | 6(30.00) | 7(18.92) |  |
| PD-L1 expression |  |  |  | 0.949 |
| ＜1 | 2(3.51) | 1(5.00) | 1(2.70) |  |
| 1-49 | 4(7.02) | 1(5.00) | 3(8.11) |  |
| ≥50 | 11(19.30) | 3(15.00) | 8(21.62) |  |
| Not examined | 40(70.18) | 15(75.00) | 25(67.57) |  |
| Cycles of treatment, median(range), No. | 5(1-23) | 8(2-23) | 5(1-18) | 0.0706 |
| Combined status |  |  |  | 0.555 |
| Single chemotherapy | 39(68.42) | 15(75.00) | 24(64.86) |  |
| Double chemotherapy | 18(31.58) | 5(25.00) | 13(35.14) |  |
| Follow up time,(range), days | 314(40-1288) | 643(68-1288) | 273(40-861) | 0.1048 |
| Overall response rate | 24(42.11) | 8(40.00) | 16(43.24) | 0.519 |

ECOG, Eastern Cooperative Oncology Group; CNS, central nervous system; EGFR, epidermal growth factor receptor gene; ALK, anaplastic lymphoma kinase gene; PD-L1, programmed cell death ligand 1; No., number.

| Table S7. Cox Proportional Hazard Regression Analysis of Progression-Free Survival on patients with NSCLC | | | | |
| --- | --- | --- | --- | --- |
|  | Univariable Hazard Ratio (95% CI) | P Value | Multivariable Hazard Ratio (95% CI) | P Value |
| pembrolizumab | 0.877(0.645-1.192) | 0.402 | 0.917(0.663-1.267) | 0.598 |
| age | 0.991(0.976-1.007) | 0.289 | 0.996(0.979-1.013) | 0.621 |
| Sex | 0.786(0.554-1.116) | 0.181 | 1.198(0.818-1.755) | 0.353 |
| ECOG performance status | 2.340(1.622-3.376) | **0.000** | 1.729(1.170-2,554) | **0.006** |
| Smoking history | 0.806(0.590-1.100) | 0.173 |  |  |
| Stage | 0.923(0.588-1.447) | 0.727 | 1.320(0.819-2.130) | 0.255 |
| CNS versus no CNS | 1.294(0.911-1.836) | 0.150 | 0.931(0.632-1.370) | 0.715 |
| Intrathoracic only versus no Intrathoracic | 1.867(1.371-2.541) | **0.000** |  |  |
| Histology | 0.823(0.598-1.132) | 0.231 | 0.802(0.561-1.148) | 0.228 |
| EGFR mutation status |  |  |  |  |
| Negative | 1 |  |  |  |
| Positive | 1.405(0.900-2.193) | 0.134 |  |  |
| Not examined | 0.832(0.590-1.175) | 0.296 |  |  |
| ALK fusion status |  |  |  |  |
| Negative | 1 |  |  |  |
| Positive | 1.014(0.322-3.191) | 0.981 |  |  |
| Not examined | 0.722(0.501-1.038) | 0.079 |  |  |
| PD-L1 expression |  |  |  |  |
| ＜1 | 1 |  | 1 |  |
| 1-49 | 1.019(0.466-2.23) | 0.962 | 1.082(0.477-2.456) | 0.851 |
| ≥50 | 0.617(0.272-1.397) | 0.247 | 0.604(0.264-1.385) | 0.234 |
| Not examined | 1.107(0.562-2.183) | 0.768 | 0.714(0.356-1.432) | 0.343 |
| Treatment lines | 2.252(1.556-3.261) | **0.000** | 2.372(1.527-3.685) | **0.000** |
| Combined with chemotherapy | 0.582(0.424-0.799) | **0.001** | 0.480(0.338-0.679) | **0.000** |
| Cycles of treatment, median(range), No. | 0.868(0.835-0.901) | **0.000** | 0.852(0.820-0.886) | **0.000** |

CI, confidence interval; ECOG, Eastern Cooperative Oncology Group; CNS, central nervous system; EGFR, epidermal growth factor receptor gene; ALK, anaplastic lymphoma kinase gene; PD-L1, programmed cell death ligand 1; No., number.

| Table S8. Cox Proportional Hazard Regression Analysis of Progression-free survival on patients with NSCLC in the first line therapy. | | | | |
| --- | --- | --- | --- | --- |
|  | Univariable Hazard Ratio (95% CI) | P Value | Multivariable Hazard Ratio (95% CI) | P Value |
| pembrolizumab | 0.986(0.490-1.984) | 0.968 | 0.886(0.641-1.224) | 0.462 |
| age | 1.002(0.966-1.04) | 0.915 | 0.991(0.975-1.008) | 0.297 |
| Sex | 0.516(0.238-1.119) | 0.094 | 1.314(0.843-2.048) | 0.229 |
| ECOG performance status | 8,745(3.091-24.75) | **0.000** | 2.045(1.383-3.025) | **0.000** |
| Smoking history | 0.485(0.245-0.961) | **0.0382** | 0.768(0.508-1.160) | 0.21 |
| Stage | 1.854(0.427-8.053) | 0.41 | 1.298(0.798-2.109) | 0.293 |
| CNS versus no CNS | 1.168(0.404-3.373) | 0.775 | 0.962(0.650-1.424) | 0.846 |
| Intrathoracic only versus no Intrathoracic | 1.080(0.542-2.149) | 0.827 |  |  |
| Histology | 1.396(0.715-2.727) | 0.329 | 0.770(0.537-1.103) | 0.154 |
| PD-L1 expression |  |  |  |  |
| ＜1 | 1 |  | 1 |  |
| 1-49 | 1.048(0.285-3.850) | 0.944 | 0.975(0.421-2.261) | 0.953 |
| ≥50 | 0.581(0.129-2.615) | 0.48 | 0.655(0.280-1.531) | 0.329 |
| Not examined | 1.132(0.331-3.868) | 0.843 | 0.883(0.440-1.770) | 0.726 |
| Combined with chemotherapy | 0.981(0.491-1.962) | 0.957 | 0.434(0.306-0.615) | **0.000** |
| Cycles of treatment, median(range), No. | 0.919(0.851-0.991) | **0.029** | 0.853(0.821-0.887) | **0.000** |

CI, confidence interval; ECOG, Eastern Cooperative Oncology Group; CNS, central nervous system; PD-L1, programmed cell death ligand 1; No., number.

| Table S9. Cox Proportional Hazard Regression Analysis of Progression-free survival on patients with NSCLC receiving PD-1 inhibitors monotherapy in the first line therapy. | | | | |
| --- | --- | --- | --- | --- |
|  | Univariable Hazard Ratio (95% CI) | P Value | Multivariable Hazard Ratio (95% CI) | P Value |
| pembrolizumab | 0.738(0.233-2.336) | 0.605 | 2.410(0.141-41.276) | 0.544 |
| age | 1.036(0.972-1.104) | 0.281 | 0.967(0.852-1.097) | 0.603 |
| Sex | 0.552(0.166-1.842) | 0.334 | 0.878(0.064-12.054) | 0.922 |
| ECOG performance status | 8.159(2.169-30.69) | **0.002** | 55.453(1.122-2740.661) | **0.044** |
| Smoking history | 0.453(0.143-1.436) | 0.179 |  |  |
| Stage | 0.758(0.097-5.957) | 0.793 | 1.817(0.081-40.621) | 0.706 |
| CNS versus no CNS | 0.648(0.082-5.094) | 0.680 | 0.192(0.003-12.385) | 0.438 |
| Intrathoracic only versus no Intrathoracic | 1.234(0.390-3.909) | 0.721 |  |  |
| Histology | 1.229(0.393-3.845) | 0.723 | 0.339(0.043-2.640) | 0.301 |
| PD-L1 expression |  |  |  |  |
| ＜1 | 1 |  | 1 |  |
| 1-49 | 2.768(0.250-30.65) | 0.407 | 1.221(0.038-39.351) | 0.910 |
| ≥50 | 0.587(0.037-9.423) | 0.707 | 0.088(0.001-8.740) | 0.301 |
| Not examined | 3.063(0.381-24.625) | 0.293 | 0.447(0.017-11.971) | 0.631 |
| Cycles of treatment, median(range), No. | 0.934(0.843-1.034) | 0.188 | 0.864(0.736-1.015) | 0.075 |

CI, confidence interval; ECOG, Eastern Cooperative Oncology Group; CNS, central nervous system; PD-L1, programmed cell death ligand 1; No., number.

| Table S10. Cox Proportional Hazard Regression Analysis of Progression-free survival on patients receiving combined therapy in the first line therapy | | | | |
| --- | --- | --- | --- | --- |
|  | Univariable Hazard Ratio (95% CI) | P Value | Multivariable Hazard Ratio (95% CI) | P Value |
| pembrolizumab | 1.247(0.458-3.397) | 0.666 | 3.494(0.904-13.507) | 0.070 |
| age | 0.980(0.936-1.026) | 0.386 | 1.012(0.954-1.075) | 0.685 |
| Sex | 0.410(0.145-1.16) | 0.093 | 0.131(0.031-0.553) | **0.006** |
| ECOG performance status | 12.01(1.237-116.6) | **0.032** | 4.835(0.291-80.253) | 0.272 |
| Smoking history | 0.453(0.143-1.436) | 0.179 |  |  |
| Stage | 2.822(0.357-22.33) | 0.326 | 23.213(1.181-456.433) | **0.039** |
| CNS versus no CNS | 1.652(0.469-5.812) | 0.434 | 1.235(0.211-7.239) | 0.815 |
| Intrathoracic only versus no Intrathoracic | 0.891(0.361-2.201) | 0.803 |  |  |
| Histology | 1.625(0.691-3.824) | 0.266 | 1.303(0.375-4.529) | 0.677 |
| PD-L1 expression |  |  |  |  |
| ＜1 | 1 |  | 1 |  |
| 1-49 | 0.283(0.052-1.530) | 0.143 | 0.693(0.057-8.500) | 0.774 |
| ≥50 | 0.226(0.029-1.746) | 0.154 | 0.283(0.019-4.097) | 0.354 |
| Not examined | 0.265(0.051-1.376) | 0.114 | 0.224(0.021-2.438) | 0.219 |
| combination status | 1.928(0.644-5.773) | 0.241 | 1.018(0.206-5.022) | 0.982 |
| Cycles of treatment, median(range), No. | 0.831(0.720-0.958) | **0.011** | 0.732(0.583-0.917) | **0.007** |

CI, confidence interval; ECOG, Eastern Cooperative Oncology Group; CNS, central nervous system; PD-L1, programmed cell death ligand 1; No., number.

| Table S11. Cox Proportional Hazard Regression Analysis of Progression-free survival on patients with NSCLC in the second line therapy. | | | | |
| --- | --- | --- | --- | --- |
|  | Univariable Hazard Ratio (95% CI) | P Value | Multivariable Hazard Ratio (95% CI) | P Value |
| pembrolizumab | 0.956(0.675-1.352) | 0.798 | 0.884(0.612-1.278) | 0.513 |
| age | 0.998(0.980-1.016) | 0.836 | 0.992(0.973-1.012) | 0.424 |
| Sex | 0.945(0.637-1.402) | 0.779 | 1.450(0.945-2.224) | 0.089 |
| ECOG performance status | 1.682(1.129-2.505) | **0.011** | 1.425(0.934-2.176) | 0.101 |
| Smoking history | 1.029(0.724-1.461) | 0.875 |  |  |
| Stage | 0.982(0.602-1.602) | 0.941 | 1.264(0.748-2.138) | 0.381 |
| CNS versus no CNS | 1.133(0.776-1.653) | 0.518 | 0.967(0.632-1.479) | 0.876 |
| Intrathoracic only versus no Intrathoracic | 1.901(1.327-2.722) | **0.000** | 1.887(1.277-2.789) | **0.001** |
| Histology | 0.784(0.538-1.141) | 0.204 | 0.735(0.476-1.136) | 0.166 |
| EGFR mutation status |  |  |  |  |
| Negative | 1 |  |  |  |
| Positive | 0.999(0.630-1.584) | 0.996 |  |  |
| Not examined | 0.757(0.508-1.129) | 0.172 |  |  |
| ALK fusion status |  |  |  |  |
| Negative | 1 |  |  |  |
| Positive | 0.780(0.247-2.465) | 0.672 |  |  |
| Not examined | 0.695(0.461-1.048) | 0.083 |  |  |
| PD-L1 expression |  |  |  |  |
| ＜1 | 1 |  | 1 |  |
| 1-49 | 1.310(0.474-3.619) | 0.603 | 1.266(0.433-3.701) | 0.666 |
| ≥50 | 0.494(0.185-1.318) | 0.159 | 0.644(0.230-1.805) | 0.403 |
| Not examined | 0.723(0.317-1.651) | 0.442 | 0.680(0.289-1.600) | 0.377 |
| Combined with chemotherapy | 0.584(0.401-0.851) | **0.005** | 0.591(0.330-0.760) | **0.001** |
| Cycles of treatment, median(range), No. | 0.845(0.807-0.884) | **0.000** | 0.836(0.798-0.877) | **0.000** |

CI, confidence interval; ECOG, Eastern Cooperative Oncology Group; CNS, central nervous system; EGFR, epidermal growth factor receptor gene; ALK, anaplastic lymphoma kinase gene; PD-L1, programmed cell death ligand 1; No., number.

| Table S12. Cox Proportional Hazard Regression Analysis of Progression-free survival on patients receiving PD-1 inhibitors monotherapy in the second line therapy. | | | | |
| --- | --- | --- | --- | --- |
|  | Univariable Hazard Ratio (95% CI) | P Value | Multivariable Hazard Ratio (95% CI) | P Value |
| pembrolizumab | 0.793(0.521-1.206) | 0.278 | 0.772(0.497-1.200) | 0.250 |
| age | 0.995(0.975-1.016) | 0.630 | 0.986(0.962-1.012) | 0.293 |
| Sex | 0.948(0.592-1.516) | 0.822 | 1.560(0.921-2.642) | 0.098 |
| ECOG performance status | 1.572(0.994-2.486) | 0.053 | 1.421(0.862-2.342) | **0.168** |
| Smoking history | 1.127(0.745-1.707) | 0.571 |  |  |
| Stage | 0.994(0.584-1.693) | 0.983 | 1.341(0.757-2.375) | 0.314 |
| CNS versus no CNS | 0.935(0.598-1.462) | 0.767 | 1.084(0.642-1.832) | 0.762 |
| Intrathoracic only versus no Intrathoracic | 1.853(1.168-2.942) | **0.009** | 1.855(1.139-3.020) | **0.013** |
| Histology | 0.755(0.484-1.179) | 0.217 | 0.779(0.468-1.294) | 0.334 |
| EGFR mutation status |  |  |  |  |
| Negative | 1 |  |  |  |
| Positive | 1.023(0.586-1.788) | 0.935 |  |  |
| Not examined | 0.858(0.534-1.379) | 0.527 |  |  |
| ALK fusion status |  |  |  |  |
| Negative | 1 |  |  |  |
| Positive | 0.564(0.137-2.314) | 0.426 |  |  |
| Not examined | 0.837(0.523-1.340) | 0.459 |  |  |
| PD-L1 expression |  |  |  |  |
| ＜1 | 1 |  | 1 |  |
| 1-49 | 0.816(0.238-2.795) | 0.746 | 1.060(0.274-4.098) | 0.933 |
| ≥50 | 0.276(0.069-1.112) | 0.070 | 0.303(0.066-1.383) | 0.123 |
| Not examined | 0.568(0.207-1.563) | 0.274 | 0.568(0.190-1.698) | 0.322 |
| Cycles of treatment, median(range), No. | 0.842(0.798-0.888) | **0.000** | 0.832(0.785-0.883) | **0.000** |

CI, confidence interval; ECOG, Eastern Cooperative Oncology Group; CNS, central nervous system; EGFR, epidermal growth factor receptor gene; ALK, anaplastic lymphoma kinase gene; PD-L1, programmed cell death ligand 1; No., number.

| Table S13. Cox Proportional Hazard Regression Analysis of Progression-free survival on patients receiving combined therapy in the second line therapy | | | | |
| --- | --- | --- | --- | --- |
|  | Univariable Hazard Ratio (95% CI) | P Value | Multivariable Hazard Ratio (95% CI) | P Value |
| pembrolizumab | 2.043(1.004-4.154) | **0.049** | 2.396(0.788-7.283) | 0.124 |
| age | 0.989(0.952-1.028) | 0.576 | 1.008(0.968-1.049) | 0.711 |
| Sex | 0.907(0.434-1.895) | 0.794 | 1.095(0.436-2.746) | 0.847 |
| ECOG performance status | 1.955(0.842-4.538) | 0.119 | 0.883(0.246-3.168) | 0.849 |
| Smoking history | 0.964(0.494-1.881) | 0.915 |  |  |
| Stage | 2.1(0.503-8.764) | 0.309 | 2.114(0.350-12.773) | 0.415 |
| CNS versus no CNS | 1.698(0.816-3.534) | 0.157 | 2.026(0.627-6.552) | 0.238 |
| Intrathoracic only versus no Intrathoracic | 1.524(0.795-2.922) | 0.205 |  |  |
| Histology | 0.783(0.386-1.586) | 0.497 | 0.652(0.253-1.684) | 0.377 |
| EGFR mutation status |  |  |  |  |
| Negative | 1 |  |  |  |
| Positive | 1.030(0.447-2.372) | 0.945 |  |  |
| Not examined | 0.594(0.276-1.280) | 0.184 |  |  |
| ALK fusion status |  |  |  |  |
| Negative | 1 |  | 1 |  |
| Positive | 2.534(0.334-19.250) | 0.369 | 1.745(0.152-20.085) | 0.655 |
| Not examined | 0.389(0.158-0.956) | **0.040** | 1.063(0.307-3.687) | 0.923 |
| PD-L1 expression |  |  |  |  |
| ＜1 | 1 |  | 1 |  |
| 1-49 | 5.994(0.877-40.965) | 0.068 | 7.703(0.660-89.920) | 0.103 |
| ≥50 | 1.073(0.226-5.091) | 0.930 | 2.179(0.402-11.804) | 0.366 |
| Not examined | 0.954(0.225-4.051) | 0.949 | 1.626(0.302-8.769) | 0.572 |
| combination status | 0.976(0.473-2.013) | 0.947 | 0.892(0.342-2.330) | 0.816 |
| Cycles of treatment, median(range), No. | 0.797(0.719-0.884) | **0.000** | 0.815(0.715-0.929) | **0.002** |

CI, confidence interval; ECOG, Eastern Cooperative Oncology Group; CNS, central nervous system; EGFR, epidermal growth factor receptor gene; ALK, anaplastic lymphoma kinase gene; PD-L1, programmed cell death ligand 1; No., number.
